# Maintaining Treatment Fidelity of Mindfulness-Based Relapse Prevention Intervention for Alcohol Dependence: A Randomized Controlled Trial Experience

**DOI:** 10.1155/2017/9716586

**Published:** 2017-07-05

**Authors:** Aleksandra E. Zgierska, Joshua Shapiro, Cindy A. Burzinski, Faith Lerner, Victoria Goodman-Strenski

**Affiliations:** ^1^School of Medicine and Public Health, Department of Family Medicine and Community Health, University of Wisconsin-Madison, 1100 Delaplaine Ct., Madison, WI 53715, USA; ^2^Mayo Clinic, Department of Medicine, Division of Internal Medicine, 200 First St SW, Rochester, MN 55905, USA; ^3^UnityPoint Health Meriter, 202 S Park St, Madison, WI 53715, USA; ^4^University Hospital and Clinics, 600 Highland Ave, Madison, WI 53792, USA

## Abstract

**Background:**

Treatment fidelity is essential to methodological rigor of clinical trials evaluating behavioral interventions such as Mindfulness Meditation (MM). However, procedures for monitoring and maintenance of treatment fidelity are inconsistently applied, limiting the strength of such research.

**Objective:**

To describe the implementation and findings related to fidelity monitoring of the Mindfulness-Based Relapse Prevention for Alcohol Dependence (MBRP-A) intervention in a 26-week randomized controlled trial.

**Methods:**

123 alcohol dependent adults were randomly assigned to MM (MBRP-A and home practice, adjunctive to usual care; *N* = 64) or control (usual care alone; *N* = 59). Treatment fidelity assessment strategies recommended by the National Institutes of Health Behavior Change Consortium for study/intervention design, therapist training, intervention delivery, and treatment receipt and enactment were applied.

**Results:**

Ten 8-session interventions were delivered. Therapist adherence and competence, assessed using the modified MBRP Adherence and Competence Scale, were high. Among the MM group participants, 46 attended ≥4 sessions; over 90% reported at-home MM practice at 8 weeks and 72% at 26 weeks. They also reported satisfaction with and usefulness of MM for maintaining sobriety. No adverse events were reported.

**Conclusions:**

A systematic approach to assessment of treatment fidelity in behavioral clinical trials allows determination of the degree of consistency between intended and actual delivery and receipt of intervention.

## 1. Introduction

Procedures to monitor and enhance treatment fidelity are the necessary methodological components of clinical trials that help ensure internal and external validity and reliability of behavioral interventions [1, 2]. Systematic implementation of strategies to maintain and optimize intervention fidelity is prerequisite for establishing credible data, drawing conclusions on intervention efficacy or effectiveness, and future replication in other studies or dissemination in clinical practice [2].

Drawing on existing evidence, guidelines, and experience of content experts, the National Institutes of Health (NIH) Behavior Change Consortium's Treatment Fidelity Workgroup [2] conceptualized fidelity as consisting of several methodological areas: strategies to address treatment integrity* (Was the study design and the intervention appropriate for the research question? Was the intervention delivered as intended?)*; strategies to assess and improve treatment receipt* (Were the participants able to understand and perform intervention taught techniques during intervention delivery?)*; and strategies to evaluate and optimize treatment enactment* (Were the participants able to apply the intervention-taught techniques in “real-life”?)*. Gearing et al. [3] further delineated core ingredients of fidelity that should be assessed as an integral part of behavioral intervention research.

Following the growing public, clinical, and scientific interest in mind-body approaches for general well-being and a variety of health problems, Mindfulness Meditation (MM) programs, often based on the Mindfulness-Based Stress Reduction (MBSR), Mindfulness-Based Cognitive Therapy (MBCT), or Mindfulness-Based Relapse Prevention (MBRP) models [4–6], have been incorporated into the clinical care for substance use disorders (SUDs) [7]. The conceptual framework described in the literature supports the application of MM for relapse prevention in SUDs and suggests that combining MM with traditional, standard-of-care cognitive behavioral therapy (CBT) techniques for SUDs may produce additive benefits [8, 9]. Although promising, research on the efficacy of MM-based interventions in SUDs is inconclusive and limited by relatively small sample sizes and heterogeneity of used methods, including a variety of targeted populations, tested interventions, and strategies for treatment fidelity monitoring [10–15]. The recent Agency for Healthcare Research and Quality (AHRQ) report called for clinical trials of MM-based interventions to rigorously incorporate both monitoring and reporting on treatment fidelity [13].

To address this gap, we conducted a 26-week randomized controlled trial (RCT; *N* = 123) evaluating efficacy of the MM-based intervention for alcohol relapse prevention and, using existing guidelines [1–3], developed and applied methods to measure and enhance the intervention's fidelity. This article describes treatment fidelity related methods and findings, which can be relevant to other behavioral intervention trials.

## 2. Methods

### 2.1. Study Design

The main study was a 26-week parallel-arm RCT assessing the efficacy of the MBRP for Alcohol Dependence (MBRP-A) intervention, adjunctive to usual care, and compared to usual care alone, for relapse prevention in alcohol dependence. The efficacy findings will be described elsewhere. The trial procedures were approved by the University of Wisconsin Health Sciences Institutional Review Board and registered with https://clinicaltrials.gov/ prior to participant enrollment.

### 2.2. Participants/Settings

Participants were alcohol dependent adults in early recovery recruited from eight local addiction treatment centers. Eligibility was determined in a two-step process, based on self-report. The initial screen was conducted by phone; those who “passed” this screen were invited for an in-person final screening, during which the extent of alcohol and drug use and related harms were assessed using the Structured Clinical Interview for DSM-IV (SCID) [18]. Eligibility criteria were age ≥ 18 years; English fluency; a SCID-confirmed diagnosis of alcohol dependence in an early remission (defined by lack of heavy drinking: men 5 drinks/day, women 4 drinks/day [19]; and lack of any drinking on ≥3 consecutive days) of 2–14 weeks; completed at least 2 weeks of outpatient treatment (≥2 therapy sessions/week) in one of the collaborating treatment centers; having a home address, phone number, and ability to reliably participate; absence of regular MM practice, preexisting bipolar or delusional disorders, current pregnancy, and a SCID-determined diagnosis of active (past 2 weeks) drug abuse or dependence; and, because stress reduction was the hypothesized mechanism of MBRP-A's action, an elevated total score (>13 points) on the 10-item Perceived Stress Scale [20, 21].

### 2.3. Randomization

The study statistician prepared the randomization envelopes (1 : 1), which were consecutively distributed to participants by the study coordinator after the baseline assessment.

### 2.4. Study Procedures

Eligible and interested individuals completed the informed consent procedures, then baseline data collection, followed by randomization (64 MM; 59 control participants) and scheduling of the MM participants for the MBRP-A course. Control participants were reminded about their eligibility to receive the intervention after completing the follow-up (wait-list controls). Outcome data were collected at baseline, and 8-week (after intervention; F1) and 26-week (F2) follow-up, and included surveys focused on substance use, drinking-related consequences and psychological health, and serum levels of stress-responsive biomarkers (interleukin-6; liver enzymes) assayed from a venous blood sample.

The study team primarily consisted of the study coordinator and the principal investigator (PI) who also functioned as the main study physician. All participants were contacted by the study coordinator prior to scheduled activities as a reminder, to facilitate protocol adherence and assist with transportation, if needed. They were also contacted by phone by the PI for adherence problems and by the study physician to discuss any concerns (e.g., intent to harm; worrisome symptoms; elevated liver enzyme levels) and were sent a letter with liver enzyme results that included a personalized note summarizing any phone conversations about abnormal findings.

### 2.5. Study Intervention

At baseline, all participants reported engaging in outpatient therapy for alcohol dependence (“usual care”) that typically included motivational enhancement, relapse prevention, and 12-step facilitation strategies [22], but not MM. They were encouraged to continue usual care per recommendations of their regular clinicians. In addition, the MM participants received the MBRP-A intervention.

With permission and assistance from MBRP's authors, the MBRP-A intervention was adapted from the existing, manualized MBRP program for SUDs [6] to address the needs of alcohol dependent adults. MBRP-A consisted of eight weekly, therapist-led, manual-driven 2-hour group sessions. It provided intensive training in MM, linking MM to CBT-based relapse prevention strategies to create a foundation for acquiring complementing MM and CBT skills for alcohol relapse prevention, as detailed elsewhere [17]. At the first session, participants received a study binder with session-specific handouts; CDs with guided formal meditations (body scan recorded by the study therapist (FL); a set of CDs with meditations by Kabat-Zinn [23]); and a meditation cushion to facilitate home practice. Each MBRP-A session followed the same format: introduction and review of participants' home practice (experiences, questions, and concerns); practice of 2–4 different techniques separated by a discussion of participant experiences and MM as a means of coping with challenges (e.g., stress, craving) that may contribute to relapse and a review of home practice recommendations for the following week (Table 1). In addition, experimental participants were asked to practice MM at home throughout the 26-week study (formal practice: 30 minutes/day, ≥6 days/week; informal practice, e.g., “mindfulness of daily activities,” “urge surfing:” daily).

### 2.6. Considerations and Measures Related to Treatment Fidelity Monitoring

Efficacy-related findings, describing the effects of the MBRP-A intervention on alcohol consumption (primary outcome, assessed retrospectively with the Timeline Followback method [24, 25]), and the severity of drinking-related consequences (secondary outcome, assessed with Drinker Inventory of Consequences [26–28]) will be presented elsewhere. The considerations and measures, detailed below, pertain to fidelity monitoring of the MBRP-A intervention.

Following the existing recommendations [2, 3], we grouped treatment fidelity related methods and measures into four categories (Table 2), addressing the core components: (1) study/intervention design so that it is rooted in a conceptual model or existing clinical practice and enables hypothesis testing; (2) standardized training of therapists to ensure appropriate implementation of the intervention; (3) monitoring and enhancement of intervention delivery so that it is implemented as intended; and (4) monitoring and improving participant understanding and performance of the taught skills (treatment receipt) and their appropriate application in real-life settings (treatment enactment).

#### 2.6.1. Study/Intervention Design

During the study development phase, a theoretical framework behind the application of MM for relapse prevention in SUDs had already been described, suggesting that MM, especially when combined with standard-of-care relapse prevention CBT strategies, can be effective for SUDs [8, 9], leading to the creation of MBRP, which leverages both MM and relapse prevention CBT techniques [6]. We adapted the MBRP program to serve the specific needs of alcohol dependent adults (MBRP-A) and tested the main hypotheses that MBRP-A will reduce the rates of alcohol relapse and the severity of drinking-related consequences. To address these aims, we considered several study designs and settled on a parallel, two equal-arm 6-month RCT comparing MBRP-A plus usual care to usual care alone; therefore, we enrolled only those who reported engagement in a treatment program for alcohol dependence. We elected to add MBRP-A to usual care, rather than assess MBRP-A as a stand-alone intervention, because alcohol dependence can have serious health consequences if left untreated and evidence-based treatments for alcohol dependence exist. This design facilitated cost-efficient data collection and efficacy analysis but led to differences in “treatment dose” (i.e., therapist contact time, group effect) across the two study arms, as only the intervention group received MBRP-A, therefore, limiting our ability to draw conclusions about “true efficacy” of MBRP-A versus nonspecific intervention effects. If shown efficacious in the present RCT, the next study could compare MBRP-A against an “active” comparison condition so that the “treatment dose” is the same across the study arms. With the aim of evaluating the effects of MBRP-A on relapse prevention, the study targeted alcohol dependent individuals in a recovery of 2–14-week duration, a period during which the risk for alcohol withdrawal is reduced while the risk for relapse is increased [29–31].

Therapists needed to have a degree in clinical psychology, social work, or substance abuse counseling; ≥2 years of experience in mental health and/or substance abuse counseling and group therapy facilitation; ≥2 years of a personal MM practice; and experience teaching MM in group settings. Several candidates were interviewed by the PI who then selected one primary therapist (FL) and one main back-up therapist (VGS).

The intervention manual for MBRP-A (available upon request) was adapted from the MBRP's curriculum [6] and provided a scripted protocol for intervention delivery. The MBRP intervention [6] included MM, with its curriculum patterned after the MBSR [4] and MBCT [5] programs, and relapse prevention CBT strategies [29, 30, 32, 33] for SUDs. At the time of our project start date, no conclusive data were available for the MBRP's efficacy. With the study focus on alcohol dependence, MBRP's manual [6] was adapted by the PI (with Alan G. Marlatt (deceased) and other authors' permission) to the needs of our study population. The PI (AZ), a family medicine and addiction medicine physician and MM practitioner, received training in the MBRP delivery from the MBRP's authors. The initial MBRP-A manual (Version 1) was pilot-tested in a 16-week uncontrolled clinical trial (*N* = 19), which showed promising results [17]. Afterwards, the manual was revised (Version 2), then retested in an 8-week uncontrolled clinical trial (*N* = 7; unpublished results), and refined further (Version 3) prior to its implementation in the RCT. All revisions were finalized by the PI and informed by the therapist and participant feedback, the PI's direct observation of the intervention delivery in both pilot trials, and expert input [6, 30, 32–34]; disagreements were resolved by consensus, with Dr. Marlatt, an expert in MM and CBT-based relapse prevention, designated to make decisions in case a consensus was not reached.

An a priori plan for potential implementation set-backs was developed. A plan for when to cancel/reschedule an intervention session was prepared. Two trained back-up therapists (VGS served as the main back-up therapist) were available to replace the primary therapist if needed. A safety protocol was developed, with all staff trained in its implementation by the PI in how to approach participants presenting with worrisome medical or mental health symptoms; the safety protocol binder, containing standardized materials, was available at each intervention session. In addition, to ensure personnel and participant safety and facilitate high-quality intervention delivery, at least two study team members (the therapist and additional research staff) were present at the intervention sessions, which were held in a quiet conference room of a centrally located community hospital with the proximity of emergency and security services. Guided by our pilot study experience [17], the sessions were scheduled in the evening to accommodate a typical work schedule, and light snacks and water were provided to improve adherence. No breaks were scheduled during the sessions; smokers were encouraged to abstain and consider cessation.

#### 2.6.2. Therapist Training

The primary therapist (FL) had 26 years of experience in mental health and substance abuse-related counseling and over 29 years of experience in MM practice, including instruction and a 7-day residential training in the MBSR delivery. The main back-up therapist (VGS) had a similar background and experience. The therapists received a protocol-driven training from the PI prior to delivering the intervention in the RCT. This training included a review of the intervention manual by the therapist, followed by a PI-led one-day, intensive workshop on MM theory, MM application to alcohol relapse prevention, and the review of the manual content and approach to intervention delivery, including role-play practices. The PI was present during the first study intervention (eight sessions) to ensure the primary therapist's competence and adherence, and provide feedback. The back-up therapist cofacilitated delivery of one intervention (eight sessions) with the primary therapist. The therapists also completed professional residential training in the delivery of MBRP offered by its developers. The primary therapist had also delivered the study interventions in two pilot trials led by the PI. In addition to the training in intervention delivery, both therapists and research staff were trained in the rationale for and implementation of the safety protocol by the PI and the research coordinator during a 2-3-hour in-person meeting.

#### 2.6.3. Intervention Delivery Monitoring

We implemented several strategies to maximize treatment fidelity. One therapist was selected to deliver all intervention sessions, with a back-up therapist available as needed to minimize the “therapist effect.” The PI observed the delivery of the first intervention (8 sessions) and was present at the beginning of each first MBRP-A session to meet with the therapist, introduce herself and the study to the participants, answer questions, encourage adherence and reporting of concerns, and thank participants for their time and effort. She was readily available to the therapist by phone and e-mail throughout the study.

*Therapist Adherence and Competence*. The therapist adherence (ability to use the strategies outlined in the intervention manual) and competence (the quality of implementation of these strategies), important components of treatment fidelity [35], were formally assessed with the modified* MBRP Adherence and Competence (MBRP-AC) scale* [36] for the delivered 10 MBRP-A courses. A trained research staff member, present at the intervention session, filled out the scale, rating the therapist adherence and competence after each session with scoring sheets submitted to the PI weekly. In addition, the intervention sessions were audio-recorded; recordings of all sessions from the first, “middle” (5th), and last (10th) interventions and two randomly selected sessions from the remaining courses were audited by a PI-trained research staff (JS) who scored therapist adherence and competence using the modified MBRP-AC scale [36]. The PI audited randomly selected individual sessions; in case of a score discrepancy between the main rater and the PI, the session was audited again and discussed until a consensus was reached. Feedback obtained through audits was communicated back to the therapist by the PI.

The original MBRP-AC scale, developed for treatment integrity monitoring of the MBRP intervention, was adapted and modified by the PI to both align its “checklist” with the content of the MBRP-A intervention and improve scoring efficiency. The audited audio-recorded sessions were scored using the modified MBRP-AC scale [36], which evaluates two session-specific dimensions of therapist performance: competence and adherence. Rating of the therapist adherence to the Key Concepts was accomplished while listening to a recording. Rating of other aspects was conducted after the session had been audited in its entirety and written notes, taken while listening to the recording, had been reviewed. For the scoring of the competence dimensions, the coder assumed a beginning score of 3 and moved to a higher or lower score from there as appropriate. If a portion of the recording was missing, preventing scoring of a given item, that item was left unscored (missing value). To enhance scoring accuracy, once all items were scored, the evaluator reaudited the session to “double-check” any suboptimal ratings or missing values.

Using the modified MBRP-AC scale, therapist* adherence* was assessed by rating the following (Table 3): (A) key treatment components, itemized in a session-specific checklist (1 = present; 0 = absent); and (B) discussion of the four Key Concepts during the session (awareness of the current experience; acceptance of the current experience; acceptance versus aversion; acceptance and action) rated on a 0 (not explained) to 3 (completely explained) scale. Therapist* competence* was evaluated by rating the following (Table 3): (A) Therapist Style/Approach in general and in the four mindfulness-related competence areas (inquiry or ability to elicit feedback and respond to verbal and nonverbal feedback; attitude or ability to model and embody the spirit of mindfulness; use of key questions or extent to which they were used to elicit discussion about practices/experiences; use of clarifying questions or extent to which the therapist addressed and clarified ideas or misconceptions about MM), rated on a 1 (absence of the desired style/approach) to 5 (consistent presence of such style/approach) scale. (B) Overall Therapist Performance in terms of the quality of intervention delivery in four areas (the overall quality of the therapy during the session; therapist/researcher ability to work as a team; therapist ability to keep the session focused and on topic; and the overall quality of MM training delivery), rated on a 1 (not satisfactory) to 5 (excellent performance) scale.

The therapist received regular feedback from the PI, stemming from the direct observations, findings from the modified MBRP-AC scoring and participant feedback throughout the study to ensure protocol competence and adherence, and prevent “therapist drift.”

#### 2.6.4. Treatment Receipt and Enactment

*Adherence to the intervention protocol* was assessed among the MM group participants by their attendance at the intervention sessions (recorded by the research staff), completion of session-specific “homework” worksheets (discussed during a subsequent session), and participant reports (logs) of home MM practice. If a participant missed a session, they received a follow-up call from the study coordinator and offered a brief (approximately 15 minutes) in-person or by-phone meeting with the therapist prior to the next session to “catch up” and encourage continued attendance and home practice. To obtain data on home MM practice, the participants were provided with “calendar” log sheets and asked to log practice minutes daily during the MBRP-A course (with logs collected weekly) and, following the MBRP-A course, to log weekly the average number of days/week and minutes/day of formal practice, and number of days/week of brief, informal MM practice, with logs collected at the follow-up assessments. These data enabled calculation of the total number of minutes of formal and times of informal practice per week. Participants were encouraged to engage in a formal practice 6 days/week, 30 minutes/day, and in informal practice daily. Participant understanding of the taught concepts and techniques (treatment receipt), and skill application (treatment enactment) were assessed through the means of a therapist-led discussion during each session about participant questions, concerns, views, and experiences, including application of the MM skills to relapse prevention and participant engagement in home practice; practicing implementation of the taught skills in hypothetical high-risk situations during the sessions; and completion and review of skill-reinforcing worksheets.

*Treatment experience and satisfaction* of the MM group participants were assessed at the last MBRP-A session by the Treatment Satisfaction Survey, developed by the research team and successfully implemented in our prior study [17]. The survey contained four questions rating a participant experience on an 11-point Likert scale (0 = “not likely at all/not important,” 10 = “very likely/very important”): (1) How important this meditation course has been to you?; (2) How useful this course has been in helping you maintain sobriety?; (3) How likely are you to continue a formal meditation practice in the future?; and (4) How likely are you to continue brief mindfulness practices in the future? At the 8-week follow-up, the MM participants also completed three Global Assessment of Treatment questions about their satisfaction with the received MBRP-A intervention (1–7 Likert scale: 1 = “extremely dissatisfied,” 7 = “extremely satisfied”); change in their alcohol problem compared to preenrollment (1–7 Likert scale: 1 = “very much worse,” 7 = “very much improved”); and helpfulness of the intervention for their alcohol problem (1–5 Likert scale: 1 = “very helpful,” 5 = “not helpful, and has made things worse”). In addition, the participants were encouraged to report side effects/adverse events at each contact point.

### 2.7. Statistical Analysis

Data were double-entered into the secure MySQL relational database and analyzed using the SPSS statistical program (version 23). Descriptive statistics were used to describe data, with results presented as a mean ± standard deviation (SD) or the number (percentage), unless indicated otherwise. The success of randomization was assessed by comparing the groups at baseline with the Mann–Whitney *U*-test for continuous data and Chi square test for categorical data; a two-tailed *p* < 0.05 was considered statistically significant.

## 3. Results

### 3.1. Study Sample

During the recruitment period (January 2010–January 2012), 292 individuals were screened. Of those, 36 declined participation, 133 were ineligible, and 123 were enrolled (64 MM, 59 control groups). Participants were, on average, 41.2 ± 11.9 years old, 90.2% Caucasian, and 43.1% female. Approximately 29.3% were unemployed and 44.7% reported an annual income <$20,000. At baseline (12 weeks prior to the quit date), participants reported 59.8 ± 34.8% of drinking days and 50.7 ± 35.3% of heavy drinking days, with 95.9% reporting heavy drinking. There were no significant differences between the groups at baseline on main outcomes, including those related to drinking (*p* values: 0.406–0.916) and drinking consequences (*p* = 0.807 for “lifetime,” and *p* = 0.056 for “past 3 months,” total scores). Outcome data were provided by 123 participants at baseline; 107 (53 MM) at F1, and 98 (47 MM) at F2. Ten (7 MM) participants withdrew from the study (91.1% retention rate), with nine reporting lack of time or a scheduling conflict and one withdrawn by the PI due to mental health-related inability to participate in study activities.

### 3.2. Intervention Delivery Monitoring

#### 3.2.1. Therapist Adherence and Competence

Over the course of the study period, 10 MBRP-A intervention cohorts (eighty sessions altogether) were delivered; 77 of these sessions were delivered by the primary therapist (FL) and 3 by the back-up therapist (VGS). Each intervention cohort included from 5 (the smallest cohort) to 18 (the largest cohort) participants. Among the 59 control group participants, 53 completed their 26-week follow-up and were able to receive the MBRP-A intervention (wait-list controls); 37 of these individuals elected to receive the intervention and participated in the MBRP-A training. The last two MBRP-A cohorts were comprised of the control group participants only.

Audio recordings of the sessions from the first, middle (5th), and last (10th) cohort (a total of 21 sessions, as the recordings of 3 sessions were unavailable due to the equipment malfunction: session 2 of cohort 4; session 8 of cohort 5; session 8 of cohort 10), and audio recordings of two randomly selected sessions from each of the remaining 7 MBRP-A cohorts (a total of 14 sessions) were audited. Therapist adherence and competence during the audited 35 sessions were scored with the modified MBRP-AC scale.

As presented in Table 3, on average, across the 35 audited sessions, the therapist adherence to the Key Treatment Component checklist, which itemized 8–10 key treatment components per session, averaged 90 ± 10%. A 100% adherence was achieved during nineteen audited sessions; 90% adherence during eleven sessions; 80% adherence during three sessions, and 70% adherence during two sessions, with lower adherence related to omitted key treatment components in Session 5 (Coping Cards, Mindful Walking in cohort 1) and Session 7 (Coping Cards, Coping Styles or Mindful Walking in cohorts 1, 5, 6, and 9). With corrective feedback, adherence increased to 90% for Session 5 in the subsequent cohorts and for Session 7 in the final cohort. Adherence to the Discussion of Key Concepts (rated on a 0–3 Likert scale) was scored at 2.8 ± 0.4 points, with the average scoring similar across the four domains (awareness of the current experience: 2.9 ± 0.4; acceptance of the current experience: 2.7 ± 0.5; acceptance versus aversion: 2.8 ± 0.5; and acceptance and action: 2.9 ± 0.4 points). Therapist competence score (1–5 Likert scale) for Style/Approach averaged 4.9 ± 0.1 points across all cohorts (Table 3), with similar scores across the four competence areas (inquiry: 5.0 ± 0.0; attitude: 5.0 ± 0.0; use of key questions: 4.9 ± 0.3; use of clarifying questions: 4.9 ± 0.3 points). The therapist Overall Performance score averaged 5.0 ± 0.1 across all cohorts, likewise with similar scores across the assessed domains (overall quality of the therapy: 4.9 ± 0.2; therapist/researcher ability to work as a team: 5.0 ± 0.0; therapist ability to keep the session focused on the topic: 4.9 ± 0.2; overall quality of MM delivery: 5.0 ± 0.0 points). Of the 35 sessions scored, only 3 sessions (one per cohorts 4, 6, and 7) did not receive maximum therapist competence scores. No trend was identified to associate lower adherence or competence scores with a specific intervention cohort or session.

### 3.3. Intervention Receipt and Enactment Monitoring

#### 3.3.1. Session Attendance

Among 64 MM group participants, 7 did not attend any intervention sessions (one withdrew from the study; one reported a scheduling conflict; five did not provide a reason) and 57 attended at least one MBRP-A session. In the latter group, 11 attended up to three sessions (with 6 attending only one session); among these participants, all but one stopped attending in the first half of the intervention; and 46 attended four or more sessions (with 22 attending seven or eight sessions).

#### 3.3.2. Home Practice

Sixty-one MM group participants provided reports on home MM practice at F1 (postintervention) and 54 provided reports at F2 (26-week follow-up). At F1, 95.1% of the reporting participants reported a formal (166.2 ± 87.7 minutes/week over 5.1 ± 2.1 days/week) and 90.2% an informal (3.6 ± 2.2 days/week) practice. At F2, 72.2% of the reporting participants reported a formal (67.1 ± 69.2 minutes/week over 2.7 ± 2.5 days/week) and 72.2% an informal (3.1 ± 2.7 days/week) practice.

#### 3.3.3. Treatment Satisfaction and Experience

(Table 4) Forty-six MM group participants completed the Treatment Satisfaction Survey at the last intervention session, rating the intervention (0–10 Likert scale) as “important” (8.2 ± 1.3) and “useful” for maintaining sobriety (7.5 ± 2.0) and stating they were likely to continue both formal (8.3 ± 1.9) and informal (8.6 ± 2.1) MM practice at home. Forty-eight MM group participants completed the Global Assessment of Treatment survey at F1 assessment, reporting the overall satisfaction with the MM intervention (5.5 ± 1.4; 1–7 Likert scale, with 7 = “extremely satisfied”), indicating that their “alcohol problem” improved since their study enrollment (5.8 ± 0.9; 1–7 Likert scale, with 7 =“very much improved”) and rating the intervention as helpful for their “alcohol problem” (1.7 ± 0.7; 1–5 Likert scale, with 1 = “very helpful”). No trend in the treatment satisfaction and experience scores was identified across the intervention cohorts.

#### 3.3.4. Intervention-Related Side Effects/Adverse Events

There were no significant concerns about participant safety during the study, and no serious or unanticipated adverse events were noted among the MM or control group participants. Only one MM participant reported a transient (resolved by 26-week follow-up) worsening of nightmares and anxiety in relation to the formal MM practice. This participant was assessed by the PI who determined the symptoms did not raise concerns for safety. Worrisome symptoms displayed by two participants during the intervention session (they were noted by other session attendees to have a smell of alcohol, without appearing intoxicated) were deemed as unlikely to be intervention-related; following the study safety protocol, these participants were assessed by the PI for disposition.

## 4. Discussion

Treatment fidelity monitoring is essential to methodological rigor of clinical trials evaluating behavioral therapies, especially complex interventions, such as MBRP-A. However, procedures for monitoring and maintenance of treatment fidelity have been inconsistently applied, limiting the strength of such research. Following the recommendations of the NIH Behavior Change Consortium [1], the present study, a 26-week RCT, systematically implemented procedures for fidelity monitoring of the MBRP-A intervention tested for alcohol relapse prevention. The study intervention was rooted in both conceptual framework and clinical practice and followed a detailed manual, fine-tuned in pilot studies. The protocol-driven approach to therapist selection and training, and the incorporation of the methods to evaluate participant treatment receipt and enactment, increased confidence that the intervention was skillfully delivered and appropriately understood and applied by the participants. The assumption of skillful intervention delivery was corroborated by the findings stemming from the measurement of the therapist competence and adherence to the treatment protocol.

Use of the existing MBRP-AC scale [36], adapted to the needs of this study, enabled a structured approach to the assessment of therapist competence and adherence, integral elements of ensuring treatment integrity [35]. Quantification of therapist adherence and competence allowed us to identify early signs of therapist skill “drift” and, through corrective feedback, to resolve this unintended variation in intervention delivery. The original and the modified MBRP-AC scales could be applied, with adaptation, to other studies of behavioral interventions, especially those based on the MBRP model, to provide a rigorous monitoring of intervention delivery.

*Limitations*. Several study design elements, which fall within the spectrum of treatment fidelity considerations, could impact our ability to draw conclusions from this study. Though self-reporting on alcohol consumption and MM home practice is the gold standard for this type of study [37, 38], the use of self-reported data can introduce a reporting/recall bias [39], especially when the recall window includes an extended period of time, such as during the final study follow-up. Although the reliance on one primary therapist to deliver the study intervention limits the therapist effect, it also makes it difficult to draw firm conclusions about the generalizability of the intervention's efficacy, as it does not allow for a separation of the therapist-specific effect from the intervention effect, and the findings will be confounded by the therapist effect in this early-stage RCT.

*Future Research*. Future research evaluating behavioral or behavior change interventions, including MM-based modalities, should strive to incorporate methods for treatment fidelity monitoring with respect to design, interventionist training, intervention delivery, receipt, and enactment. Although the specifics of a given study/intervention design as well as financial, personnel, and other constraints may determine which of the recommended procedures are appropriate and feasible, the development of treatment fidelity procedures should start during the study development stage.

## 5. Conclusions

A systematic approach to assessment of treatment fidelity in behavioral clinical trials allows determination of the degree of consistency between intended and actual delivery and receipt of therapy.

## Figures and Tables

**Table 1:** Mindfulness-Based Relapse Prevention for Alcohol Dependence (MBRP-A) Intervention: session summary.

Session	Content summary
*Week 1* Automatic pilot and relapse	Introduction to mindfulness meditation and mindfulness-based relapse prevention; being “present” versus on “autopilot”; relapse and mindfulness *Practice*: Raisin exercise; breath meditation; body scan

*Week 2* Awareness of triggers and craving	Common challenges in meditation practice; awareness of reactions to triggers and the tendency to judge our experiences; mindful response to relapse triggers, cravings, and urges *Practice*: Body scan; walking down the street exercise; urge surfing exercise; abbreviated mountain meditation

*Week 3* Mindfulness in daily life	Use of brief mindfulness techniques in daily-life situations; awareness of feelings and sensations that can arise in body and mind, including those related to craving and urges *Practice*: Mindful hearing (or “seeing”) exercise; sitting meditation (breath and body); mindful walking; SOBER minimeditation

*Week 4* Staying present and aware (mindful) in high-risk situations	Awareness of individual high-risk situations and sensations, emotions, and thoughts; being “present” and mindful during uncomfortable sensations, emotions, and thoughts *Practice*: Sitting meditation (breath, body, sounds, thoughts); SOBER minimeditation in a high-risk situation; mindful stretching

*Week 5* Balancing acceptance and skillful, mindful action (change)	Acceptance of unpleasant states of mind and body; acceptance of self; coping with problematic interpersonal interactions *Practice*: Sitting meditation with Rumi's poem; SOBER minimeditation exercise; mindful walking or mindful stretching

*Week 6* Are thoughts facts?	The role of thoughts and their relationship to relapse; understanding that thoughts are only thoughts and may not reflect facts; the difference between lapse and relapse; individual unhealthy thought patterns that may lead to relapse *Practice*: Sitting meditation (thoughts); mindful stretching

*Week 7* Self-care and life balance	Person-specific, early warning signs of relapse; coping behaviors; relapse prevention action plan; the importance of self-care and life balance, forgiveness and compassion for health and relapse prevention *Practice*: Lovingkindness meditation (or “Let go of struggle” meditation); mindful walking

*Week 8* Balanced living: building support networks, continuing to live mindfully	Life balance and mindfulness meditation as a way to maintain life balance; importance and creation of support networks; barriers to reaching out for help; reflection on the received training and ways to sustain mindfulness meditation practice; looking forward *Practice*: Body scan, concluding guided meditation

Adapted with permission from [6]; SOBER = stop, observe, breathe, expand, and respond.

**Table 2:** Core Components of treatment fidelity-related assessment and enhancement methods.

Treatment fidelity: core components	Recommended elements	Implementation of the recommended elements in the study
(1) Study/intervention design: rooted in a conceptual model or existing clinical practice and enabling hypothesis testing		

Theoretical framework	Conceptual background	Theoretical framework supporting MM as a therapy for substance use disorders was published [8, 9, 12, 16] The study intervention was adapted from the existing program developed for adults with substance use disorders [6]
	Intervention goals	Reduction of alcohol relapse (primary aim) and drinking-related consequences (secondary aim)
	Comparison arms (i) Treatment dose across the study arms (ii) Treatment dose within each arm	MM + usual care versus usual care alone (wait-list control) (i) Assessment activities were the same across the two arms; treatment dose was not equal (only the MM group received the intervention) (ii) Adherence to treatment protocol was promoted with the goal of maximum treatment dose among the MM group participants
	Participant characteristics	Alcohol-dependent adults in early (2–14 weeks) recovery, engaged in the outpatient treatment (≥2 weeks) for alcohol dependence

Therapist, team, environment characteristics	Therapist characteristics	Background in mental health and substance abuse-related counseling, with experience in applying behavioral therapies for addictive disorders; personal MM practice, including instruction
	Team structure	At least two team members (therapist + research staff) were present at each intervention session to monitor and enhance participant adherence and safety of the personnel and participants, collect data, and assist the therapist
	Environment	The intervention was delivered in a large, quiet hospital-based conference room (central location; convenient, free street parking)

Manual development	Program/session model	The intervention was patterned after MBRP [6], which, in turn, was patterned after MBCT [5] and MBSR [4]; it consisted of eight weekly two-hour therapist-led group sessions
	Minimum dose	Attendance of at least 4 intervention sessions
	Corrective feedback	Experience from two consecutive uncontrolled pilot trials (*N* = 19 [17]; and *N* = 7) and expert input allowed to refine the methods, including the intervention manual, prior to implementing them in the RCT

Planning for the implementation setbacks	Back-up therapist	A second therapist was selected and trained to be available as back-up for the primary therapist
	Session rescheduling	Protocol was developed in advance for when to cancel/reschedule the intervention sessions (e.g., inclement weather)
	Safety protocol	All research personnel were trained by the PI in the safety protocol steps, outlined in a binder present at each session, in case of worrisome medical or mental health symptoms in the study participants; at least two study team members needed to be present at each session; the sessions were held in a hospital conference room guaranteeing a proximity of the emergency and security services

(2) Training of the therapists: ensuring appropriate implementation of the intervention		

Therapist Training	Standardized training	Primary therapist was trained by the PI according to the intervention manual, then delivered the intervention in two pilot trials. She then received additional protocol-driven, day-long training from the PI prior to delivering the intervention in the RCT; the first study intervention (eight sessions) was directly observed by the PI. The back-up therapist received a protocol-driven training and then cofacilitated delivery of one intervention (eight sessions) with the primary therapist The protocolized training included a didactic portion on alcohol addiction, relapse prevention, and mindfulness meditation; manual-driven review and discussion of each session's content; and role-playing; the therapists created flash cards outlining each session's main points to enhance adherence to the intervention manual
	Booster training/certification	The therapists completed additional formal training (5-day residential course) in MBRP, offered by its developers

(3) Monitoring and enhancement of intervention delivery: ensuring it is implemented as intended		

Control for provider differences	Therapist effect	One (primary) therapist delivered the intervention during the whole study; a second (back-up) therapist was available as needed
Adherence to treatment protocol	Therapist adherence and competence	The therapist adherence and competence were scored with the modified MBRP-AC scale: (a) after each intervention session by the researcher present at the session; and (b) by a PI-trained auditor who audited selected audio-recorded sessions; all intervention sessions were audio-recorded
Corrective feedback	Feedback on therapist adherence and competence	Suboptimal scores of the MBRP-AC scale were discussed with the therapist by the PI
	Participant feedback	Participant feedback on the intervention content, delivery, and settings was actively sought throughout the study; no modifications to the intervention protocol were needed as based on this feedback

(4) Intervention receipt and enactment monitoring: monitoring and improving participant understanding and performance of the taught skills and their appropriate application in real-life settings		

Treatment adherence	Session attendance	Strategies to promote participant adherence to session attendance: reminder phone calls; transportation assistance; scheduling of the intervention sessions in late afternoon to accommodate typical work schedule; snacks at the intervention sessions; reaching out by the study coordinator to those who missed a session
	Home practice	Strategies to promote participant home practice: discussion at the intervention sessions of barriers, facilitators and experiences related to MM practice

Treatment receipt	Understanding of the concepts taught during the sessions	Inquiry by the therapist about questions, comments or problems at each session; therapist-facilitated discussion among the session participants about session-specific core topics and a review session-specific home practice
	Ability to use the taught skills	Linking of the taught skills to relapse prevention during each session; practicing implementation of the taught skills in hypothetical high-risk situations during the session; discussion and review of the skill implementation at home

Treatment enactment	Ensure use of the taught skills in appropriate life settings	Discussion and review of the skills and their application to specific situations at home; logs of home practice; survey on treatment satisfaction and experiences at the last intervention session; Global Assessment of Treatment survey at the 8-week follow-up visit

MBCT: Mindfulness-Based Cognitive Therapy; MBRP: Mindfulness-Based Relapse Prevention; MBRP-AC: Mindfulness-Based Relapse Prevention Adherence and Competence scale; MBSR: Mindfulness-Based Stress Reduction; MM: Mindfulness Meditation.

**Table 3:** Therapist adherence and competence across the intervention sessions and cohorts, as measured by the modified MBRP-AC scale.

	Overall	Session 1	Session 2	Session 3	Session 4	Session 5	Session 6	Session 7	Session 8
*Therapist adherence*									

*(A) Key Treatment Components* ^1^									
mean ± SD	0.9 ± 0.10	1.0 ± 0.0	1.0 ± 0.1	1.0 ± 0.0	0.9 ± 0.1	0.8 ± 0.1	1.0 ± 0.0	0.8 ± 0.1	1.0 ± 0.1
C1–10: score for a given session in the assessed cohort	C1: 0.9 ± 0.1 C2: 1.0 ± 0.0 C3: 1.0 ± 0.0 C4: 0.9 ± 0.1 C5: 0.9 ± 0.1 C6: 0.9 ± 0.1 C7: 0.9 ± 0.1 C8: 0.9 ± 0.1 C9: 0.9 ± 0.2 C10: 0.9 ± 0.1	C1: 1.0 C3: 1.0 C5: 1.0 C8: 1.0 C10: 1.0	C1: 0.9 C5: 0.9 C9: 1.0 C10: 1.0	C1: 1.0 C5: 1.0 C7: 1.0 C10: 1.0	C1: 0.9 C2: 1.0 C4: 0.9 C5: 0.9 C6: 1.0 C10: 0.9	C1: 0.8 C5: 0.9 C8: 0.9 C10: 0.9	C1: 1.0 C4: 1.0 C5: 1.0 C10: 1.0	C1: 0.7 C5: 0.8 C6: 0.8 C9: 0.7 C10: 0.9	C2: 1.0 C3: 1.0 C7: 0.9

*(B) Discussion of Key Concepts* ^2^									
mean ± SD	2.8 ± 0.4	2.8 ± 0.3	2.9 ± 0.3	3.0 ± 0.0	2.9 ± 0.1	3.0 ± 0.0	2.9 ± 0.1	2.9 ± 0.3	2.2 ± 0.9
C1–10: score for a given session in the assessed cohort	C1: 2.9 ± 0.1 C2: 2.1 ± 1.2 C3: 2.3 ± 0.0 C4: 2.8 ± 0.2 C5: 2.9 ± 0.3 C6: 3.0 ± 0.0 C7: 3.0 ± 0.0 C8: 3.0 ± 0.0 C9: 3.0 ± 0.0 C10:2.9 ± 0.1	C1: 3.0 C3: 2.3 C5: 3.0 C8: 3.0 C10: 2.8	C1: 2.8 C5: 3.0 C9: 3.0 C10: 2.8	C1: 3.0 C5: 3.0 C7: 3.0 C10: 3.0	C1: 3.0 C2: 3.0 C4: 2.8 C5: 2.8 C6: 3.0 C10: 2.8	C1: 3.0 C5: 3.0 C8: 3.0 C10: 3.0	C1: 2.8 C4: 3.0 C5: 3.0 C10: 3.0	C1: 3.0 C5: 2.3 C6: 3.0 C9: 3.0 C10: 3.0	C2: 1.3 C3: 2.3 C7: 3.0

*Therapist competence*									

*(A) Style/ approach* ^3^									
mean ± SD	4.9 ± 0.1	5.0 ± 0.1	4.9 ± 0.1	5.0 ± 0.0	5.0 ± 0.0	5.0 ± 0.0	5.0 ± 0.0	5.0 ± 0.1	4.8 ± 0.1
C1–10: score for a given session in the assessed cohort	C1: 5.0 ± 0.0 C2: 4.8 ± 0.4 C3: 4.8 ± 0.0 C4: 5.0 ± 0.0 C5: 5.0 ± 0.0 C6: 5.0 ± 0.0 C7: 4.8 ± 0.4 C8: 4.9 ± 0.2 C9: 5.0 ± 0.1 C10 2.9 ± 0.1	C1: 5.0 C3: 4.8 C5: 5.0 C8: 5.0 C10: 5.0	C1: 5.0 C5: 5.0 C9: 5.0 C10: 4.8	C1: 5.0 C5: 5.0 C7: 5.0 C10: 5.0	C1: 5.0 C2: 5.0 C4: 5.0 C5: 5.0 C6: 5.0 C10: 5.0	C1: 5.0 C5: 5.0 C8: 5.0 C10: 5.0	C1: 5.0 C4: 5.0 C5: 5.0 C10: 5.0	C1: 5.0 C5: 5.0 C6: 5.0 C9: 4.8 C10: 5.0	C2: 5.0 C3: 4.8 C7: 4.8

*(B) Overall performance* ^4^									
mean ± SD	5.0 ± 0.1	4.9 ± 0.2	5.0 ± 0.0	5.0 ± 0.0	5.0 ± 0.0	5.0 ± 0.0	4.9 ± 0.1	5.0 ± 0.1	5.0 ± 0.0
C1–10: score for a given session in the assessed cohort	C1: 5.0 ± 0.0 C2: 5.0 ± 0.0 C3: 4.8 ± 0.4 C4: 4.9 ± 0.2 C5: 5.0 ± 0.1 C6: 5.0 ± 0.0 C7: 5.0 ± 0.0 C8: 5.0 ± 0.0 C9: 5.0 ± 0.0	C1: 5.0 C3: 4.5 C5: 5.0 C8: 5.0 C10: 5.0	C1: 5.0 C5: 5.0 C9: 5.0 C10: 5.0	C1: 5.0 C5: 5.0 C7: 5.0 C10: 5.0	C1: 5.0 C2: 5.0 C4: 5.0 C5: 5.0 C6: 5.0 C10: 5.0	C1: 5.0 C5: 5.0 C8: 5.0 C10: 5.0	C1: 5.0 C4 4.8 C5: 5.0 C10: 5.0	C1: 5.0 C5: 5.0 C6: 5.0 C9: 4.8 C10: 5.0	C2: 5.0 C3: 5.0 C7: 5.0

C – Cohort; MBRP-AC - Mindfulness-Based Relapse Prevention Adherence and Competence scale;  ^1^Score range for the presence of Key Treatment Components: 1 = the checklist item was addressed; 0 = the checklist item was not addressed;  ^2^Score range for Discussion of Key Concepts for each of the four assessed domains (awareness of the current experience; acceptance of the current experience; acceptance versus aversion; acceptance and action): 3 = concept was discussed in detail and completely explained, 2 = discussed with some explanation but not as thoroughly or opportunities to discuss it further may have been missed, 1 = only briefly mentioned, 0 = not discussed;  ^3^Score range for Therapist Style/Approach in general and for the mindfulness-related competence in four areas (inquiry or ability to elicit feedback and respond to verbal and nonverbal feedback; attitude or ability to model and embody the spirit of mindfulness; use of key questions or extent to which they were used to elicit discussion about practices/experiences; use of clarifying questions or extent to which the therapist addressed and clarified ideas or misconceptions about MM): 5 = the therapist consistently demonstrated desired style/approach; 4 = during most but not the whole session; 3 = for approximately half of the session; 2 = infrequently; 1 = absence of such style/approach;  ^4^Score range for Overall Performance for each of the four assessed domains (the overall quality of the therapy; therapist/researcher ability to work as a team; therapist ability to keep the session focused on the topic; the overall quality of MM delivery): 5 = the overall performance was “excellent,” consistent with the manual; 4 = was “good,” with only minor deficiencies in a small portion of the session; 3 = was “satisfactory,” consistently less than excellent; 2 = was “mediocre,” consistently less than satisfactory; 1 = was “not satisfactory,” with poor performance throughout the session.

**Table 4:** Treatment experience and satisfaction among the intervention group participants during the study.

Measure	Overall	Cohort 1	Cohort 2	Cohort 3	Cohort 4	Cohort 5	Cohort 6	Cohort 7	Cohort 8
*Treatment Satisfaction Survey (completed at the last intervention session; N = 46)*									

Importance of the meditation course^1^	8.2 ± 1.3	7.4 ± 2.0	7.8 ± 0.8	8.0 ± 1.0	9.2 ± 1.0	8.7 ± 0.8	8.5 ± 1.9	8.0±1.6	7.5 ± 0.6
Usefulness of the course in helping maintain sobriety^1^	7.5 ± 2.0	7.4 ± 2.1	8.0 ± 1.6	7.1 ± 2.1	8.0 ± 1.8	7.7 ± 1.2	6.3 ± 3.3	7.7 ± 2.7	8.0 ± 1.4
Likelihood to continuing a formal meditation practice in the future^1^	8.3 ± 1.9	7.8 ± 2.3	8.8 ± 1.3	7.7 ± 2.6	9.2 ± 0.8	9.0 ± 0.9	8.0 ± 2.2	7.7 ± 2.5	8.5 ± 1.7
Likelihood of continuing brief mindfulness practices in the future^1^	8.6 ± 2.1	8.8 ± 2.2	7.0 ± 3.3	8.4 ± 2.3	9.7 ± 0.5	9.7 ± 0.8	8.5 ± 2.4	8.7 ± 1.4	7.5 ± 3.0

*Global Assessment of Treatment (completed at the 8-week follow-up; N = 48)*									

Satisfaction with the received MM therapy^2^	5.5 ± 1.4	6.0 ± 0.8	5.0 ± 2.2	5.6 ± 1.0	4.8 ± 1.8	6.2 ± 0.4	5.6 ± 2.1	6.0 ± 0.9	5.2 ± 1.4
Overall change in alcohol problem since starting the study^3^	5.8 ± 0.9	6.0 ± 0.0	5.8 ± 0.5	5.6 ± 0.8	5.8 ± 0.4	5.8 ± 0.8	6.4 ± 0.9	5.1 ± 0.8	5.8 ± 0.9
Helpfulness of the MM program for alcohol problem^4^	1.7 ± 0.7	1.8 ± 0.5	1.4 ± 0.5	1.6 ± 0.5	1.7 ± 0.5	1.7 ± 0.5	1.4 ± 0.5	1.8 ± 0.7	1.7 ± 0.7

^1^Responses on a 0–10 Likert scale: 0 = not likely at all/not important, 10 = very likely/very important;  ^2^Responses on a 1–7 Likert scale: 1 = extremely dissatisfied, 7 = extremely satisfied;  ^3^Responses on a 1–7 Likert scale: 1 = very much worse, 7 = very much improved;  ^4^Responses on a 1–5 Likert scale: 1 = very helpful, 5 = not helpful and has made things worse.
